# Supplementary material for: Clear amniotic fluid aspiration syndrome: A novel description of an old entity
Source: PLoS One. 2024 Jun 10;19(6):e0301595. doi: 10.1371/journal.pone.0301595 (PMC11164380; doi:10.1371/journal.pone.0301595)
Supplement: Attachment — Submitted filename: Answer to reviewers comments.docx [file pone.0301595.s001.docx]

**Reviewers' comments:**

Reviewer's Responses to Questions

**Comments to the Author**

1. Is the manuscript technically sound, and do the data support the conclusions?

The manuscript must describe a technically sound piece of scientific research with data that supports the conclusions. Experiments must have been conducted rigorously, with appropriate controls, replication, and sample sizes. The conclusions must be drawn appropriately based on the data presented.

Reviewer #1: No

Reviewer #2: Yes

2. Has the statistical analysis been performed appropriately and rigorously?

Reviewer #1: N/A

Reviewer #2: Yes

3. Have the authors made all data underlying the findings in their manuscript fully available?

The [PLOS Data policy](http://www.plosone.org/static/policies.action#sharing) requires authors to make all data underlying the findings described in their manuscript fully available without restriction, with rare exception (please refer to the Data Availability Statement in the manuscript PDF file). The data should be provided as part of the manuscript or its supporting information, or deposited to a public repository. For example, in addition to summary statistics, the data points behind means, medians and variance measures should be available. If there are restrictions on publicly sharing data—e.g. participant privacy or use of data from a third party—those must be specified.

Reviewer #1: No

Reviewer #2: Yes

4. Is the manuscript presented in an intelligible fashion and written in standard English?

PLOS ONE does not copyedit accepted manuscripts, so the language in submitted articles must be clear, correct, and unambiguous. Any typographical or grammatical errors should be corrected at revision, so please note any specific errors here.

Reviewer #1: No

Reviewer #2: Yes

5. Review Comments to the Author

Please use the space provided to explain your answers to the questions above. You may also include additional comments for the author, including concerns about dual publication, research ethics, or publication ethics. (Please upload your review as an attachment if it exceeds 20,000 characters)

Reviewer #1: The authors carried out a retrospective study of full-term infants, born between 2001 and 2022, who at birth presented respiratory symptoms and radiological signs possibly suggestive of aspiration of amniotic fluid (clear or meconium-stained). The aim would be to focus the attention of the public on a new and, according to the authors, not uncommon pathology called clear amniotic fluid aspiration syndrome.

**PREAMBLE TO THE ANSWERS TO REVIEWER 1. PROFESSOR SIMONE PRATESI.**

Dear colleague, I am (PYR) 69 years old (40 years of NICU) and still « Emeritus Pratician » in my neonatal department in the Maternal and Pediatric Hospital in the University of South-Réunion (a maternity with 4,000 births per year), where I continue to go twice a week to continue my resesarch (Perinatal Epidemiology, preeclampsia, gestational weight gain and fœtal outcomes).

I began my internship on the 1st of October 1980 in the university maternity of Pointe-à-Pitre, Guadeloupe, French West Indies. At this time, the doxa in my department was to intubate **ALL newborns (100%)** with meconium-stained liquids (and it was the specific duty of interns). We were supposed to follow the Gregory’s famous 1976 study on intubation for thick meconium-stained newborns. During my 4 years of intership, the incidence of MS deliveries was 17% (80% of babies from African-American origin). Therefore, during these 4 years (2,500 births per year there), WE HAVE INTUBATED 1,700 term babies, 90% of them having APGAR 10. As you say perfectly below : *« The history of meconium aspiration syndrome tells us that an invasive procedure, such as tracheal aspiration, was introduced into the clinical routine before there was scientific evidence of its usefulness »,* and I have lived (days and nights) during 4 yeaars this strangeness.

Being intern, I was obliged to obey, and I should have done in 4 years 500 such intubations ( !!) even if did not believe that an APGAR 10 newborn was ill, and as you say also below *«  Intubation can be a simple procedure for experienced physicians but it remains an invasive procedure for the patient* ».

I became a full practitioner in 1986, and I STOPPED intubating these babies AGAINST THE INTERNATIONAL RECOMMENDATIONS OF THE TIME (see the « TALE » reported by our Chinese colleagues, reference 1. As a matter of fact , in my mind, recommendations concerning meconium-stained births have been the biggest cumulative errors of the young history-40 years- of neonatology).

Then I went in 1991-1993 to do a 13 months Post-Doc of perinatal epidemiology in Charleston, South-Carolina, USA where they have also a lot of African-American babies.

The result was :

**Alexander GR, Hulsey TC, Robillard PY, De Caunes F, Papiernik E. Determinants of meconium-stained amniotic fluid in term pregnancies. J Perinatol. 1994 Jul-Aug;14(4):259-63. PMID: 7965219.**

Where we showed that African newborns had three times more meconium-stained than Caucasian ones only because they were most mature (neglected post-maturity), and not that they were 3 times more “hypoxic stressed”.

I arrived at the University of Réunion in 1999 and established the Perinatal database on January the 1st, 2001. We did as you say « *The authors carried out a retrospective study of full-term infants* », but all the data have been recorded « au fil de l’eau » prospectively in fact day after day (the coding always done by a neonatologist concerning each newborn, the maternal part filled by a midwife).

In our 23-year database (2001-2023), and I became the chief of the NICU department in 2002 until 2014, the rate of intubation at birth in our term babies :

- MECONIUM-STAINED deliveries (N= 12,999) : TWO percents (2.0%). In my personal professional life, I have lived a rate of 100% intubation in meconium stained deliveries during my internship to 2% when I was Chief of the NICU( !!!).
- Clear amniotic fluid (N= 66,823): ZERO point 4 (0.4%)

But, in these intubated babies, we could also notice that some of them were « drowned » with even a geyser like jet at the intubation, necessitating a minimum of liquid suction (MS as well as clear amniotic fluids). Our work is to give our clinical experience and report that aspiration may also exist in clear liquids.

**Paste your Logo here**

**Biography.** Neonatologist, epidemiologist, specialist- in tropical diseases and perinatal epidemiology. 44 **y**ears of work in tropical countries (French overseas departments and Territories): 16 years in Guadeloupe (1979-1995, French West-Indies, Caribbean’s), 3 years in Tahiti (1995-1998, French Polynesia, Pacific) and 24 years (since 1999) in Reunion, French overseas Department, Indian Ocean. All his career in level 3 NICU’s and university hospitals. Twelve years (2002-2014) at the head of the neonatal department and NICU of the University hospital South Reunion (Saint-Pierre). Currently working at Centre Hospitalier Universtaire Sud-Réunion, Saint-Pierre.

Creator of the Reunion perinatal epidemiological database (2001), and the International Workshop on Immunology of Preeclampsia (since 1998), and co-founder of the Centre d’Epidémiologie périnatale Océan Indien CEPOI (2010). One year of post-Doctoral Fellowship in perinatal epidemiology (MUSC, Medical University of South Carolina, Charleston, USA, 1991-1992). International course of Epidemiology CDC Atlanta (1992).

**ANSWERS TO REVIEWER 1.**

Unfortunately, the proposed study has too many methodological limits to be able to reach the fixed objective.

We have chosen to compare our experience Clear amniotic fluid aspiration with the worst situation : meconium fluid aspiration in term babies to show that our described situation is also (while much less) a significant problem. This « syndrome » being completely neglected, very often these ditressed newborns are fighting for breathing in thircraddle in the maernity ward hours after birth before finding any help.

All sections of the paper have a lot of limitations. For example, the proposed diagnosis of clear amniotic fluid aspiration syndrome is very questionable; it would require an “… evident liquid suction at the endotracheal intubation (in the delivery room), productive suction of less than 15 minutes…”.

We have added in Methods, page 4 :

“In those (a great minority, 0.4%) who have been intubated in the delivery room because of clinical safety necessity (non-vigorous newborn with bad APGAR score and/or with constant bilateral crackling lung sounds at auscultation, severe Silverman score) a productive suction of less than 15 minutes was coined as mild aspiration.

Does this mean that all newb »orns with breathing effort at birth should be intubated to allow a tracheal suction? Intubation can be a simple procedure for experienced physicians but it remains an invasive procedure for the patient,

Not at all Sir, in our maternity, only 2.0% of our meconium-stained newborns are intubated in the delivery room and 0.4% of our clear amniotic fluids. This is why we have added in the sentence :

“non-vigorous newborn with bad APGAR score and/or with constant bilateral crackling lung sounds at auscultation, severe Silverman score”

to be performed only if scientific evidence has proven its usefulness. The history of meconium aspiration syndrome tells us that an invasive procedure, such as tracheal aspiration, was introduced into the clinical routine before there was scientific evidence of its usefulness.

We totally agree with you. We did not perform because of proven scientific evidence. All the intubated babies were

« In those (a great minority, 0.4%) … have been intubated in the delivery room because of clinical safety necessity”

And yet, such scientific evidence is missing in meconium-stained fluid, and this is the reason why International guidelines for neonatal resuscitation since 2015 suggest against tracheal aspiration of newborns from meconium-stained amniotic fluid deliveries.

We have rewritten page 13 :

**Management in the delivery room of CAF-AS**. Besides the current debates in the interest of endotracheal intubation with suction of the meconium fluid in the delivery room in MSAF-AC [10-14] (but recommendations in the MSAF-AS have so many times changed in the last 3 decades, see the “tale” [1]), for example, now in International guidelines for neonatal resuscitation since 2015 suggest against tracheal aspiration of newborns from meconium-stained amniotic fluid deliveries [1]. However, in the current special case of clear fluids we are reporting, we wish to promote a different debate. Clear liquids being very fluid, they are very easy to suction at birth. We have even many experiences when we perform endotracheal intubation in these babies to have the surprise to face a real initial geyser. . Further studies could be to 1) investigate among midwives and anesthesiologists before a misunderstood newborns with breathing effort if something happened during the long course of labor (in particular long-lasting hypotensive events in the mother). This would permit to quantify exactly this cause of CAF-AS 2) In this particular case (clear amniotic fluid), could an immediate suction at birth be useful as compared with non-intervention ?

The proposed procedure “In our experience, a repeated sequence of [passive oxygenation- cautious thoracic clapping-suction] until the total disappearance of bilateral crackling lung sounds leads to a quasi-recovery of the problem” cannot be acceptable because it lacks scientific foundation and does not take into account the pathophysiology of postnatal respiratory adaptation.

WE HAVE COMPLETELY DELETED THE SENTENCE :

“In our experience, a repeated sequence of [passive oxygenation- cautious thoracic clapping-suction] until the total disappearance of bilateral crackling lung sounds leads to a quasi-recovery of the problem”

Reviewer #2: The paper investigated an important concept which commonly gives misinformation on clinical practice. As the author concluded , the terminology Clear amniotic fluid aspiration syndrome is not existent and it rarely happens which could be due to expulsive labor in multigravida's. The message which was written on the discussion and conclusion part has to be clear.

We have rewritten the conclusions :

**“The two major specificities of this CAF-AS are, first, hyperkinetic explosive deliveries in multiparas and, second, long-lasting (8-10 minutes) episodes of maternal hypotension due to epidural/spinal anaesthesia occurring sometimes even one hour before expulsion. Future studies, independent from the 40-year huge debates on management of meconium-stained aspiration syndrome in the delivery room, this specific entity (clear amniotic fluid) may benefit of a specific management at birth. For example, before an evidently “drowned like” baby, severe respiratory distress, bilateral crackling lung sounds, intubation at birth could be considered (these clear liquids being very easy to suction).”**

We thank the reviewers for their positive critics.
