# Supplementary material for: Clear amniotic fluid aspiration syndrome: A novel description of an old entity
Source: PLoS One. 2024 Jun 10;19(6):e0301595. doi: 10.1371/journal.pone.0301595 (PMC11164380; doi:10.1371/journal.pone.0301595)
Supplement: Attachment — Submitted filename: review.docx [file pone.0301595.s002.docx]

**Plos-One Points for Review**

- What are the main claims of the paper and how significant are they for the discipline?
  - *The claims of the study are that a new and never-reported-before entity referred to as clear amniotic fluid aspiration exists. Another claim is that this entity is less severe than MFAS.*
- Are the claims properly placed in the context of the previous literature? Have the authors treated the literature fairly?
  - *Improvement needed in the structure of the discussion*
- Do the data and analyses fully support the claims? If not, what other evidence is required?
  - *As stated below, there are claims not supported by the results.*
- *PLOS ONE* encourages authors to publish detailed protocols and algorithms as supporting information online. Do any particular methods used in the manuscript warrant such treatment? If a protocol is already provided, for example for a randomized controlled trial, are there any important deviations from it? If so, have the authors explained adequately why the deviations occurred?
  - *The authors can provide a protocol for clear amnioticfluid aspiration.*
- If the paper is considered unsuitable for publication in its present form, does the study itself show sufficient potential that the authors should be encouraged to resubmit a revised version?
  - *Yes, after heavy scientific editing it can become a very interesting and novel report*
- Are original data deposited in appropriate repositories and accession/version numbers provided for genes, proteins, mutants, diseases, etc.?
  - *Not relevant*
- Does the study conform to any [relevant guidelines](https://journals.plos.org/plosone/s/best-practices-in-research-reporting#loc-reporting-guidelines-for-specific-study-types) such as CONSORT, MIAME, QUORUM, STROBE, and the [Fort Lauderdale agreement](https://www.sanger.ac.uk/wp-content/uploads/fortlauderdalereport.pdf)?
- Are details of the methodology sufficient to allow the experiments to be reproduced?
  - *No, needs a definition of the condition and of the outcomes assessed.*
- Is any software created by the authors freely available?
  - *Not relevant*
- Is the manuscript well organized and written clearly enough to be accessible to non-specialists?
- *Not in it’s current form*
- Is it your opinion that this manuscript contains an [NIH-defined experiment of Dual Use concern](https://grants.nih.gov/grants/guide/notice-files/NOT-OD-13-107.html)?
  - *No*

General comment- I am very enthusiastic for the manuscript since this is an important entity which neonatologists are aware of but hasn’t been officially reported. Therefore the subject is both novel and clinically important. However, in my opinion it needs professional scientific editing to be taken seriously by readers. I will lay out my suggestions for the abstract to make my point.

The title of the manuscript is too long. I suggest something like- Clear amniotic fluid aspiration syndrome: a novel description of an old entity.

Abstract

The introduction of both the abstract and the manuscript shouldn’t contain any of the study’s’ findings. You should only present what’s known in the literature.

I would say that the study’s’ aims were: 1. to characterize the risk factors and outcomes associated with Clear Amniotic Fluid Aspiration Syndrome and 2. to compare the outcomes of Clear Amniotic Fluid Aspiration to Meconium Aspriration.

Methods- Is not professionally worded. It’s grammatically flawed to begin a sentence with a numerical number. I would suggest beginning traditionally: This was an observational study of….over a 22 year period in a single medical center. Compared were parturient/labor characteristics and neonatal outcomes in cases with suspected Clear Amniotic Fluid Aspiration to cases suspected for Meconium Aspiration.

Results

Of 79,620 term deliveries there were 66,705 (83.8%) clear amniotic fluids and 12,915 (16.2%) meconium stained amniotic fluid (MSAF) ; Of neonates born with clear amniotic fluid, 166 (0.25%) were diagnosed with Clear Amniotic Fluid Aspiration Syndrome (CAF-AS) While 202 (15.7%) of those born with MSAF, were diagnosed with aspiration syndrome (MSAF-AS). Both conditions had comparable rates of mild manifestation (% vs%, p=). Persistent pulmonary hypertension (PPH) occurred 5 times less in CAF-AS than MSAF-AS ( OR vs OR, p <) Both conditions presented similar rates of surfactant destruction without PPH (% vs %, p=). There was 1 postnatal death in CAF-AS vs 10 in MSAF-AS.

Conclusion

CAF-AS were quite similar in terms of numbers to MSAF-AS (166 vs 202). We identified in these cases two major specific causes: hyperkinetic explosive deliveries in multiparas and long-lasting episodes of maternal hypotension due to epidural/spinal anaesthesia during labour. Out of 140 million births per year in the world, it should concern 3 million neglected cases nowadays. Future studies should evaluate if this CAF-AS should benefit from a more active intervention such as immediate endotracheal suction at birth, this clear fluid being very easy to suction.

Reviewer comment: The conclusion states findings that aren’t presented in the results, such as the hyperkinetic explosive deliveries and the maternal hypotension. Also, the first sentence is misleading. It refers to the number of cases in the study, and compares it between the clear amniotic fluid aspiration and meconium aspiration. For a meaningful comparison, you need to compare the prevalence and not the number. If the denominator is the number of term deliveries for both conditions, then they will have a similar prevalence.

Introduction

Like the rest of the manuscript, the introduction lacks scientific professional editing. Here are the main comments:

-The first sentence needs citing of references.

-The use of parenthesis and punctuation marks to introduce thoughts is not accepted in scientific papers. I refer to the following sentence:

“Hence, MSAF-AS may be probably the topic that induced the

highest controversies [3] if not errors of judgment (« tales » ?).”

-Use of questions to make a point, is not accepted in scientific papers such as:

“Should we perform endotracheal intubation at birth in the delivery room, and to whom? Should we suction the liquid coming from the lung? Vigorous on non-vigorous babies?”

-The aim of the study should be clear and concise. Currently the paragraph intended to lay out the aims of the study is a long paragraph that could be summarized to say:

CAF-AS is a condition never reported before in the literature. We aim to describe associated risk factors and outcome and compare it to meconium aspiration syndrome.

Methods

All the relevant information is present. But scientific editing is significantly needed. Please write the criteria needed for the diagnosis of CAF-AS. Also add the outcomes assessed.

Statistical analysis section is well written.

Ethical approval section is too long and cumbersome.

Results

Scientific editing is required for the text and the tables. The statistics seem fine. I am not familiar with the term “explosive delivery” that appears in the conclusion, nor do I see this or maternal hypotension, in the results section, both of which appear in the conclusion. You can’t state in the conclusion findings that do not appear in the results

Discussion

Scientific editing is heavily required. What exactly is the point of figure 1? The discussion usually doesn’t have figures or tables.

To sum up, the manuscript addresses a novel subject. The methods and analyses are fine. However, the manuscript requires heavy scientific editing to become publishable.
