# Supplementary material for: Clear amniotic fluid aspiration syndrome: A novel description of an old entity
Source: PLoS One. 2024 Jun 10;19(6):e0301595. doi: 10.1371/journal.pone.0301595 (PMC11164380; doi:10.1371/journal.pone.0301595)
Supplement: Attachment — Submitted filename: Answer to 2nd REVISION reviewer 4.docx [file pone.0301595.s003.docx]

**Plos-One Points for Review**

- What are the main claims of the paper and how significant are they for the discipline?
  - *The claims of the study are that a new and never-reported-before entity referred to as clear amniotic fluid aspiration exists. Another claim is that this entity is less severe than MFAS.*
- Are the claims properly placed in the context of the previous literature? Have the authors treated the literature fairly?
  - *Improvement needed in the structure of the discussion*
- Do the data and analyses fully support the claims? If not, what other evidence is required?
  - *As stated below, there are claims not supported by the results.*
- *PLOS ONE* encourages authors to publish detailed protocols and algorithms as supporting information online. Do any particular methods used in the manuscript warrant such treatment? If a protocol is already provided, for example for a randomized controlled trial, are there any important deviations from it? If so, have the authors explained adequately why the deviations occurred?
  - *The authors can provide a protocol for clear amnioticfluid aspiration.*
- If the paper is considered unsuitable for publication in its present form, does the study itself show sufficient potential that the authors should be encouraged to resubmit a revised version?
  - *Yes, after heavy scientific editing it can become a very interesting and novel report*
- Are original data deposited in appropriate repositories and accession/version numbers provided for genes, proteins, mutants, diseases, etc.?
  - *Not relevant*
- Does the study conform to any [relevant guidelines](https://journals.plos.org/plosone/s/best-practices-in-research-reporting#loc-reporting-guidelines-for-specific-study-types) such as CONSORT, MIAME, QUORUM, STROBE, and the [Fort Lauderdale agreement](https://www.sanger.ac.uk/wp-content/uploads/fortlauderdalereport.pdf)?
- Are details of the methodology sufficient to allow the experiments to be reproduced?
  - *No, needs a definition of the condition and of the outcomes assessed.*
- Is any software created by the authors freely available?
  - *Not relevant*
- Is the manuscript well organized and written clearly enough to be accessible to non-specialists?
- *Not in it’s current form*
- Is it your opinion that this manuscript contains an [NIH-defined experiment of Dual Use concern](https://grants.nih.gov/grants/guide/notice-files/NOT-OD-13-107.html)?
  - *No*

**DETAILED REVIEW (Reviewer 4). Modifications in red in the text**

General comment- I am very enthusiastic for the manuscript since this is an important entity which neonatologists are aware of but hasn’t been officially reported. Therefore the subject is both novel and clinically important. However, in my opinion it needs professional scientific editing to be taken seriously by readers. I will lay out my suggestions for the abstract to make my point.

We thank the reviewer for his positive critics and the suggestions which make the manuscript much more readable and synthetized. In fact the title and the abstract are completely re-written by him. Thank you.

1. **The title** of the manuscript is too long. I suggest something like- Clear amniotic fluid aspiration syndrome: a novel description of an old entity. E

The title is now as suggested

2. **Abstract**

The introduction of both the abstract and the manuscript shouldn’t contain any of the study’s’ findings. You should only present what’s known in the literature.

I would say that the study’s’ aims were: 1. to characterize the risk factors and outcomes associated with Clear Amniotic Fluid Aspiration Syndrome and 2. to compare the outcomes of Clear Amniotic Fluid Aspiration to Meconium Aspriration.

This sentence “The study’s’ aims were: 1. to characterize the risk factors and outcomes associated with Clear Amniotic Fluid Aspiration Syndrome and 2. to compare the outcomes of Clear Amniotic Fluid Aspiration to Meconium Aspriration” is now in “Background”

Methods- Is not professionally worded. It’s grammatically flawed to begin a sentence with a numerical number. I would suggest beginning traditionally: This was an observational study of….over a 22 year period in a single medical center. Compared were parturient/labor characteristics and neonatal outcomes in cases with suspected Clear Amniotic Fluid Aspiration to cases suspected for Meconium Aspiration.

Methods has been reworded: “This was an observational study over a 22	year period in a single level 3 medical center. Compared were	parturient/labor characteristics and neonatal outcomes in cases with suspected Clear Amniotic Fluid Aspiration to cases suspected for Meconium Aspiration.”

Results

Of 79,620 term deliveries there were 66,705 (83.8%) clear amniotic fluids and 12,915 (16.2%) meconium stained amniotic fluid (MSAF) ; Of neonates born with clear amniotic fluid, 166 (0.25%) were diagnosed with Clear Amniotic Fluid Aspiration Syndrome (CAF-AS) While 202 (15.7%) of those born with MSAF, were diagnosed with aspiration syndrome (MSAF-AS). Both conditions had comparable rates of mild manifestation (% vs%, p=). Persistent pulmonary hypertension (PPH) occurred 5 times less in CAF-AS than MSAF-AS ( OR vs OR, p <) Both conditions presented similar rates of surfactant destruction without PPH (% vs %, p=). There was 1 postnatal death in CAF-AS vs 10 in MSAF-AS.

Results are now: Of 79,620 term deliveries there were 66,705 (83.8%) clear amniotic fluids and 12,915 (16.2%) meconium stained amniotic fluid (MSAF) ; Of neonates born with clear amniotic fluid, 166 (0.25%) were diagnosed with Clear Amniotic Fluid Aspiration Syndrome (CAF-AS) While 202 (15.7%) of those born with MSAF, were diagnosed with aspiration syndrome (MSAF-AS). Both conditions had comparable rates of mild manifestation (67.5% vs 69.2%, p= 0.63). Persistent pulmonary hypertension (PPH) occurred 5 times less in CAF-AS than MSAF-AS (4% vs 20%, OR 0.17, P< 0.0001) Both conditions presented similar rates of surfactant destruction without PPH (11.1 % vs 13.4%, p= 0.87). There was 1 postnatal death in CAF-AS vs 10 in MSAF-AS.

Conclusion

The conclusion now is “CAF-AS were quantitatively quite similar in terms of need of actual active intervention of the neonatologists in the delivery room (166 vs 202, i.e. in terms of numbers of cases and not prevalence) to MSAF-AS We identified in these cases two major specific causes: hyperkinetic explosive deliveries in multiparas and long-lasting episodes of maternal hypotension due to epidural/spinal anaesthesia during labour. Out of 140 million births per year in the world, it should concern 3 million neglected cases nowadays. Future studies should evaluate if this CAF-AS should benefit from a more active intervention such as immediate endotracheal suction at birth, this clear fluid being very easy to suction.”

Reviewer comment: The conclusion states findings that aren’t presented in the results, such as the hyperkinetic explosive deliveries and the maternal hypotension.

They are not yet presented in results but in the discussion. As the entity being supposed not to exist, nobody was obliged to write down any special observation or suggestions in these kind of records

Also, the first sentence is misleading. It refers to the number of cases in the study, and compares it between the clear amniotic fluid aspiration and meconium aspiration. For a meaningful comparison, you need to compare the prevalence and not the number. If the denominator is the number of term deliveries for both conditions, then they will have a similar prevalence.

We have then precised now; “CAF-AS were quantitatively quite similar in terms of need of actual active intervention of the neonatologists in the delivery room (166 vs 202, i.e. in terms of numbers of cases and not prevalence) to MSAF-AS.”

I our mind, it is important that neonatologist clinicians (and especially the Junior ones) REALIZE that in terms of actual work (i.e. NUMBERS and not prevalence), they have to take care equally for both entities which was absolutely not the case the last 2 decades.

In results, we show clearly that the CAF-AS are highly underestimated (rate of no intubations and primary intubations while secondary intubations are OR 1.7, p almost significant 0.09). This means that we let these poor babies “rowing” alone with respiratory distress during long hours before being relieved.

Introduction

Like the rest of the manuscript, the introduction lacks scientific professional editing. Here are the main comments:

-The first sentence needs citing of references.

We have put 8 references now

-The use of parenthesis and punctuation marks to introduce thoughts is not accepted in scientific papers. I refer to the following sentence:

“Hence, MSAF-AS may be probably the topic that induced the

highest controversies [3] if not errors of judgment (« tales » ?).”

-Use of questions to make a point, is not accepted in scientific papers such as:

“Should we perform endotracheal intubation at birth in the delivery room, and to whom? Should we suction the liquid coming from the lung? Vigorous on non-vigorous babies?”

The paragraph : “Hence, MSAF-AS may be probably the topic that induced the highest controversies [3] if not errors of judgment (« tales » ?) in the short history of the new science “neonatology” (5-6 decades). Should we perform endotracheal intubation at birth in the delivery room, and to whom? Should we suction the liquid coming from the lung? Vigorous on non-vigorous babies? “

Has been completely deleted

-The aim of the study should be clear and concise. Currently the paragraph intended to lay out the aims of the study is a long paragraph that could be summarized to say:

CAF-AS is a condition never reported before in the literature. We aim to describe associated risk factors and outcome and compare it to meconium aspiration syndrome.

Your last sentence replaces now the original one

Methods

All the relevant information is present. But scientific editing is significantly needed. Please write the criteria needed for the diagnosis of CAF-AS. Also add the outcomes assessed.

We have added:

The criteria needed for the diagnosis of CAF-AS: severe respiratory distress in the newbornat birth (silverman score > 3), non-vigorous hypoxic baby needing tracheal intubation, abundant tracheal production of amniotic fluid at suction in the delivery room (and careful timing of this production). Bilateral crackling lung sounds **.**Need of oxygen inducing a transfer in the neonatal department (passive oxygen, n-cpap, intubation). Radiological aspects at chest X ray similar to that of MSAF-AS.

Outcomes assessed.: hours or days of oxygenation, hours or days of n-cpap or intubated mechanical ventilation with oxygen, destruction of surfactant, persistent pulmonary hypertension, death.

Statistical analysis section is well written.

Ethical approval section is too long and cumbersome.

It is now “This study was conducted in accordance with French legislation. As per new French law applicable to trials involving human subjects, a specific approval of an ethics committee is not required for this non-interventional study with anonymized data” .

Results

Scientific editing is required for the text and the tables. The statistics seem fine. I am not familiar with the term “explosive delivery” that appears in the conclusion, explosive has been replaced by rapid

nor do I see this or maternal hypotension, in the results section, both of which appear in the conclusion. You can’t state in the conclusion findings that do not appear in the results

We have added a paragraph in results: **Clinical conditions associated with CAF-AS**. The entity CAF-AS being supposed not to exist, nobody was obliged to write down any special observation or suggestions in these kind of records. Therefore, in this paragraph that we will develop inthe discussion. In these non-protocoled observations, we may recall three main entiteis : First, what we may call “acute events” at birth such as difficult and long extraction (forceps, vacuum, spatulas), prolonged foetal bradycardia (10 mn), shoulder dystocia, abruption placenta, maternal septic shock, foetal acute anaemia. the two other features which seem to be very specific to the CAF-AS are, second, hyperkinetic rapid deliveries in multiparas (“champagne cork” deliveries) and, third, long-lasting (8-10 minutes) episodes of maternal hypotension due to epidural/spinal anaesthesia (occurring even 30 minutes before expulsion).

Discussion

Scientific editing is heavily required. What exactly is the point of figure 1? The discussion usually doesn’t have figures or tables.

We have put all the Figures (chest X-Rays) in results, letting the Editor to choose where he will put them in the final article

To sum up, the manuscript addresses a novel subject. The methods and analyses are fine. However, the manuscript requires heavy scientific editing to become publishable.

All the manuscript has been revised by a CAMBRIGE PROOFREADING expert
