# Supplementary material for: Clear amniotic fluid aspiration syndrome: A novel description of an old entity
Source: PLoS One. 2024 Jun 10;19(6):e0301595. doi: 10.1371/journal.pone.0301595 (PMC11164380; doi:10.1371/journal.pone.0301595)
Supplement: Attachment — Submitted filename: second review.docx [file pone.0301595.s004.docx]

Abstract

What is surfactant destruction and how was this diagnosed?

Please display the percentage for postnatal death, currently you state the absolute number 1 vs 10.

The conclusion is unacceptable in its current form. It is not supported by the results presented in the abstract. It introduces new findings not mentioned in the results. Also, its sentences should be more concise, laying out insights with minimal use of words.

There are not enough keywords. “Suction” is nonspecific, use the commonly used term to make it clear that its endotracheal suction. I am not a neonatologist, so look up the accepted term.

Main Text

*Introduction*

Poorly written. This is not clean scientific language which is more objective and neutral. Currently, it has a lot of opinions and descriptive language in it, portraying the feelings of the author.

*Methods*

There is no methods section. The study design is not described. The definitions are a good start. Make sure to define every term needed. The definitions should have a reference if they are internationally accepted.

Results

The authors introduce terms that need clarification, like- primary/secondary intubation.

The language used is still non-scientific, for example- : “neonates were kept for dozens of minutes (even hours)”… an accurate mean time should be presented instead of a rough verbal estimation.

Or “CAF-AS were rather a problem of multiparous deliveries” instead of writing that there was a higher prevalence of multiparity in the CAFS group compared to the MAS group….

The figures are not clear If they are a description of the different cases, then present them in a table. Figures are usually a graphic description or image, not a list of case descriptions.

In the results you display the number of minutes the babies were suctioned, but in the methods you do not detail that suctioning was timed in all cases.

There are many more examples of flawed methodology and unprofessional scientific writing.

Discussion

The discussion is mostly based on the author’s experience and not on scientific evidence.

The limitation of imperfect study methodology and unprofessional scientific writing is prominent in this section as it was in previous sections.

I would suggest the authors to reorganize the data, improve the display of data, make sure every parameter is well defined and then get assistance on scientific writing, to maximize the potential of this manuscript. In my opinion, it is unpublishable in its current form. I hope the authors will succeed in doing this and getting this paper published because the subject itself seems very important clinically.
