# Supplementary material for: Clear amniotic fluid aspiration syndrome: A novel description of an old entity
Source: PLoS One. 2024 Jun 10;19(6):e0301595. doi: 10.1371/journal.pone.0301595 (PMC11164380; doi:10.1371/journal.pone.0301595)
Supplement: Attachment — Submitted filename: Response reviewer 4.docx [file pone.0301595.s005.docx]

We thank the reviewer for his positive critics and the suggestions which make the manuscript much more readable and synthetized. Modifications are in red in the text

Abstract

What is surfactant destruction and how was this diagnosed?

You are right, destruction of surfactant is an interpretation. We replaced destruction of surfactant by “need of surfactant” in all the paper. In meconium aspiration syndrome, it has been recommended for 2 decades to try surfactant therapy in these babies although they are all at term (and it works). Neonatologists interpret this as “destruction of surfactant” or “disorientation of surfactant” due to the mechanical aggression of the inhaled fluid.

Please display the percentage for postnatal death, currently you state the absolute number 1 vs 10.

This has been done and the odds-ratios calculated

The conclusion is unacceptable in its current form. It is not supported by the results presented in the abstract. It introduces new findings not mentioned in the results.

Also, its sentences should be more concise, laying out insights with minimal use of words.

The conclusion now is: “CAF-AS were quantitatively quite similar in terms of need of actual active intervention of the neonatologists in the delivery room (166 vs 202, i.e. in terms of numbers of cases and not prevalence) to MSAF-AS We identified in these cases two major specific causes: hyperkinetic explosive deliveries in multiparas and long-lasting episodes of maternal hypotension due to epidural/spinal anaesthesia during labour. Out of 140 million births per year in the world, it should concern 3 million neglected cases nowadays. Future studies should evaluate if this CAF-AS should benefit from a more active intervention such as immediate endotracheal suction at birth, this clear fluid being very easy to suction.”

There are not enough keywords. “Suction” is nonspecific,

Keywords are now: Meconium aspiration syndrome, clear amniotic fluid aspiration syndrome, endotracheal suction

use the commonly used term to make it clear that its endotracheal suction. I am not a neonatologist, so look up the accepted term.

We changed everywhere in the text the term “suction” by endotracheal suction

Main Text

*Introduction*

Poorly written. This is not clean scientific language which is more objective and neutral. Currently, it has a lot of opinions and descriptive language in it, portraying the feelings of the author.

The intoduction is now:

Meconium-stained amniotic fluid aspiration syndrome MSAF-AS has been particularly well reported for 5 decades as it may lead in newborns to severe respiratory distress syndrome with a high morbidity and mortality rate [1-7}. The terror induced by this syndrome is precisely magnified by the fact that these severe features occur in term neonates. A recent paper by Huang, Zhu and Cheng [7] comparing practices between China and the USA in the past 60 years is probably one of the best recalling exhaustively decades all the debates concerning management of MSAF-AS. International recommendations are very well synthetized in a table. These authors summarize all the neonatal resuscitation’s international recommendations: 1987, 1994, 2000, 2005-2006, 2011, 2016, and 2020 (some of them contradicting the preceding). Monfredini et al. [2] also recall very well all these evolutions and controversies [2].

“Clear amniotic fluid aspiration syndrome” CAF-AS does not yet exist in PubMed search. Typing such research gives 56 results ALL of them speaking of meconium staining (because of the root “amniotic fluid”) except one: “Suctioning of clear amniotic fluid at birth: A systematic review” [8], but the authors speak only of suctioning the mouth and nose at birth. If we type “meconium-stained amniotic fluid”, we have 1188 results.

CAF-AS is a condition never reported before in the literature. We aim to describe associated risk factors and outcomes and compare them to meconium aspiration syndrome.

*Methods*

There is no methods section. The study design is not described. The definitions are a good start. Make sure to define every term needed. The definitions should have a reference if they are internationally accepted. The reference internationally accepted is reference 3

We have added:

“The criteria needed for the diagnosis of CAF-AS: severe respiratory distress in the newbornat birth (silverman score > 3), non-vigorous hypoxic baby needing tracheal intubation, abundant tracheal production of amniotic fluid at suction in the delivery room (and careful timing of this production). Bilateral crackling lung sounds **.**Need of oxygen inducing a transfer in the neonatal department (passive oxygen, n-cpap, intubation). Radiological aspects at chest X ray similar to that of MSAF-AS.

Outcomes assessed.: hours or days of oxygenation, hours or days of n-cpap or intubated mechanical ventilation with oxygen, destruction of surfactant, persistent pulmonary hypertension, death.”

Results

The authors introduce terms that need clarification, like- primary/secondary intubation.

We have added in methods (page 4): “Primary intubation means intubation at birth directly in the delivery room. Secondary intubation means that the newborn has been intubated in the neonatal NICU department after transfer, therefore minutes or hours after birth”

The language used is still non-scientific, for example- : “neonates were kept for dozens of minutes (even hours)”… an accurate mean time should be presented instead of a rough verbal estimation.

We rephrased the sentence by “newborns with respiratory distress syndromes after a long stay at the maternity ward (even hours) before a transfer decision in the neonatal department.”, page 5

Or “CAF-AS were rather a problem of multiparous deliveries” instead of writing that there was a higher prevalence of multiparity in the CAFS group compared to the MAS group….

We have replaced (page 5) by your suggested sentence. “It is of note that there was a higher prevalence of multiparity in the CAF-AS group (while it has long been known that the MAS-AS is largely associated with primiparities), p= 0.01.”

The figures are not clear If they are a description of the different cases, then present them in a table. Figures are usually a graphic description or image, not a list of case descriptions.

Again, with all my respect, I think that all these chest X-rays are indispensable to CONVINCE NEONATOLOGISTS. They show (at a lesser extent) typical images encountered in the meconium aspiration syndrome that neonatologists know by heart. Without these, 80% of them will continue to ignore this syndrome.

In the results you display the number of minutes the babies were suctioned, but in the methods you do not detail that suctioning was timed in all cases.

In the methods, we have added (page 4).” All pediatrical interventions in the delivery room (intubation or not) were systematically timed by initializing the infant resuscitation table’s chronometer to evaluate the duration of intervention and/or the timing of intubation”

There are many more examples of flawed methodology (please see the first paragraph of the chapter below) and unprofessional scientific writing.

Discussion

The discussion is mostly based on the author’s experience and not on scientific evidence.

- With all my respect, it could not be another way, because IT IS YET AN UNKNOWN SYDROME. Therefore, it was not possible to write a specific protocol for an inexistent disease. I just copy the comment of reviewer 3: “General comment- I am very enthusiastic for the manuscript since this is an important entity which neonatologists are aware of but hasn’t been officially reported. Therefore the subject is both novel and clinically important.”
- 1) We have added in the introduction “CAF-AS is a condition never reported before in the literature. We aim to describe associated risk factors and outcome and compare it to meconium aspiration syndrome.”, page 3
- 2) We have added a paragraph in results: “**Clinical conditions associated with CAF-AS**. The entity CAF-AS being supposed not to exist, nobody was obliged to write down any special observation or suggestions in these kind of records. Therefore, in this paragraph that we will develop inthe discussion. In these non-protocoled observations, we may recall three main entities : First, what we may call “acute events” at birth such as difficult and long extraction (forceps, vacuum, spatulas), prolonged foetal bradycardia (10 mn), shoulder dystocia, abruption placenta, maternal septic shock, foetal acute anaemia. the two other features which seem to be very specific to the CAF-AS are, second, hyperkinetic rapid deliveries in multiparas (“champagne cork” deliveries) and, third, long-lasting (8-10 minutes) episodes of maternal hypotension due to epidural/spinal anaesthesia (occurring even 30 minutes before expulsion).”

The limitation of imperfect study methodology and unprofessional scientific writing is prominent in this section as it was in previous sections.

We have asked to a CAMBRIGE PROOFREADING expert

to revise the entire manuscript

I would suggest the authors to reorganize the data, improve the display of data, make sure every parameter is well defined and then get assistance on scientific writing, to maximize the potential of this manuscript. In my opinion, it is unpublishable in its current form. I hope the authors will succeed in doing this and getting this paper published because the subject itself seems very important clinically.
